# Eating Quality of Australian Grass and Grain-Fed Lamb Equally Rated by US Consumers

**DOI:** 10.3390/foods13010026

**Published:** 2023-12-20

**Authors:** Maddison T. Corlett, Liselotte Pannier, Graham E. Gardner, Andrea J. Garmyn, Mark F. Miller, David W. Pethick

**Affiliations:** 1 Australian Cooperative Research Centre for Sheep Industry Innovation, Armidale, NSW 2350, Australia; l.pannier@murdoch.edu.au (L.P.); g.gardner@murdoch.edu.au (G.E.G.); d.pethick@murdoch.edu.au (D.W.P.); 2 Centre for Animal Production and Health, Murdoch University, Murdoch, WA 6150, Australia; 3 Department of Food Science and Human Nutrition, College of Agriculture & Natural Resources, Michigan State University, East Lansing, MI 48824-1046, USA; garmynan@msu.edu; 4 Department of Animal and Food Sciences, Texas Tech University, Lubbock, TX 79409, USA; mfmrraider@aol.com

**Keywords:** sheep, diet, aging, consumer, sensory, ruminants

## Abstract

Anecdotal suggestions that US consumers perceive Australian sheepmeat as more “gamey” or “stale” compared to US sheepmeat are potentially attributable to the extended chilled shipping times contributing to longer-aged meat and predominately pasture-fed grazing systems. This study evaluated the impact of diet and extended storage times on Australian sheepmeat using sensory scores as assessed by US consumers. Meat samples from Australian sheep (*n* = 80) fed a grass or grain diet were aged in a vacuum at 1–2 °C for 5, 21 or 45 days. Untrained consumers (*n* = 960) at Texas Tech University (Lubbock, Texas) assessed samples for overall liking, tenderness, juiciness and flavour using a scale from 1 (worst) to 100 (best). In general, US consumers scored grain- and grass-fed samples within the same storage period similarly (*p* > 0.05). Furthermore, storage from 5 to 21 days improved sensory scores by a maximum of 28.6 for tenderness for grass-fed outside cuts (*p* < 0.05), while storage for 21 to 45 days did not improve eating quality for most cuts of both diets (*p* > 0.05). This is an interesting finding for the Australian sheepmeat industry as long storage time has no negative effect on eating quality and US consumers enjoyed grass- and grain-fed sheepmeat equally.

## 1. Introduction

Australia is the world’s largest exporter of sheepmeat [1], with the United States (US) as the second largest importer of Australian sheepmeat [1,2]. Sheepmeat remains a niche and unfamiliar protein for US consumers [3], but there is an increasing willingness to try sheepmeat [4]. The average per capita consumption of sheepmeat for US consumers is 0.4 kg per year per person, which is considerably lower compared to Australian consumers at 5.9 kg [5]. To validate or refute anecdotal evidence that suggests US consumers perceive Australian sheepmeat as “gamey” or “stale” required a structured and systematic approach. The majority of the Australian flock is pasture-fed, with grain feeding during the feed shortages in summer and autumn. This can result in the presence of “pastural” flavours evident in the meat of pasture-fed animals when cooked [6]. Therefore, the US consumer perceptions of sheepmeat may be based on a combination of factors including grass feeding, long storage times under chilled shipping conditions, lack of familiarity with the product, taste concerns and a lack of knowledge on how to cook and prepare sheepmeat [4]. Previous work by O’Reilly et al. [7] identified that untrained US and Australian consumers score Australian sheepmeat eating quality similarly; however, the study only tested loin and topside meat cuts and did not test animal diet differences.

Sheepmeat exported from Australia is generally stored in vacuum packaging for 3 to 8 weeks below 4 °C while being transported [8]. This extended storage period may be contributing to the lower eating quality anecdotally reported by US consumers. Increased post-mortem aging through vacuum packaging has been shown to improve the tenderness of sheepmeat from day 1 to day 12 [9] and for up to 14 days for beef [10,11]. Yet, the sensory scores of sheepmeat are reduced with aging between 21 and 42 days [12]. Therefore, storing sheepmeat under vacuum packaging beyond 21 days may have a negative impact on sensory scores.

In addition to extended storage times, the diet of the animal prior to slaughter may have a subsequent effect on sensory scores. Australian consumers tasting lamb [13] and New Zealand consumers tasting beef samples [14] could not differentiate the meat derived from grass- or grain-fed systems. In comparison, Japanese consumers have demonstrated a sensitivity to the chemical compounds in meat from grass- or grain-fed beef samples [14]. Likewise, trained French consumer panellists perceived meat from grass-fed sheep as more liver-flavoured and less tender and juicy than meat from stall-fed lambs that were fed a commercial concentrate and hay diet [15]. Differences between study findings may be due to the familiarity and habituation of Australian and New Zealand consumers to sheepmeat flavours, which may have resulted in the insensitivity of sensory preferences between products derived from grain- and grass-fed lambs [13,14]. Whereas, consumers that are not accustomed to sheepmeat flavours, such as Japanese or US consumers, may be more sensitive to the chemical compounds from grass- or grain-fed diets affecting sheepmeat eating quality [12,14], thereby supporting the anecdotal claims. For this reason, we evaluated grain- and grass-fed diets of Australian lambs and these samples were then stored for 5, 21 or 45 days prior to subsequent cooking and sensory analysis using untrained US consumers. We hypothesised that US consumers would score meat from grain-fed lamb higher than that from grass-fed lamb. We also hypothesised that 5- and 21-day-aged samples would have higher consumer eating quality scores than 45-day-aged samples. 

## 2. Materials and Methods

### 2.1. Experimental Design

Data were collected from 80 lambs from a commercial flock based at the Struan Research Centre Farm, South Australia. The lambs were single-born castrated males from Poll Dorset sires mated to Border Leicester × Merino dams. Lambs were allocated to either grain- (*n* = 40) or grass-finished (*n* = 40) diets and balanced for live weight (grass initial live weight 39.7 ± 3.37 kg; grain 39.0 ± 2.63 kg). Within each diet, lambs were allocated to one of three replicates (each with 12 to 14 lambs in each replicate). Grass-fed lambs were placed in one of three plots of mixed ryegrass and sub-clover under pivot irrigation that contained ad libitum green feed throughout the duration of the trial (Table 1). Grain-fed lambs were placed in three replicate small feedlot pens with access to lick feeders containing a recommended commercial grain-based ration and supplemented with straw as a source of roughage. After an acclimatisation period of three weeks, 25% barley was introduced to the grain ration. After a further two-week adjustment period, the ration was changed to 50% barley and 50% lupins (Table 1). The feed information in Table 1 was provided via near-infrared spectroscopy (Foss 500, Rockwall, TX, USA) following the Association of American Feed Control Officials [16] laboratory methods. All lambs were finished from weaning (approximately 3 months of age) until slaughter (approximately 6 months of age). Live weights were measured every three weeks and rations were adjusted to ensure that a target average live weight of 60 kg was achieved by all lambs (grass final live weight 59.7 ± 3.90 kg; grain 60.8 ± 4.42 kg). The lambs remained within each replicate for the duration of the feeding phase and then were mixed within a diet group during transportation and slaughter. The day prior to slaughter, the lambs were held in yards for six hours and then weighed and transported to a commercial abattoir. The lambs were then rested overnight in lairage, and all lambs were slaughtered the following day. 

### 2.2. Sample Collection and Carcass Measures

At a commercial abattoir, the lambs were slaughtered using electrical head stunning followed by exsanguination. Within one hour of slaughter, hot carcass weight and GR tissue depth (11 cm distal from the backbone, over the 12th rib) were measured for each carcass. Carcasses were electrically stimulated with a medium voltage system [17] and trimmed according to AUS-MEAT specifications [18]. The pH decline and ultimate pH were measured on the left *M. longissimus lumborum* as described by Pearce et al. [19]. In brief, this involved four pH and temperature measurements of each carcass: immediately after slaughter at ~35 °C, followed by a measure at ~20 °C, ~12 °C and the ultimate pH at 24 h post-mortem [19]. A linear regression was then utilised to estimate the predicted temperature when the pH first equalled 6 [17]. After the carcasses were chilled at 3–4 °C for 24 h, nine cuts were then collected per carcass for subsequent eating quality assessment. Both *M. longissimus lumborum* were excised between the 12th/13th rib and the caudal end of the *M. longissimus lumborum* (loin; AUSMEAT 5150). Both *M. semimembranosus*, cap off, were also excised from the carcass as a whole (topside; AUSMEAT 5077). Both *M. gluteus medius* had the tail/flank and the cap muscle removed (rump; AUSMEAT 5074). Both *M. biceps femoris* were prepared from the silverside with the heel muscle removed along the natural seam (outside; AUSMEAT 5075). The small muscle size of the *M. gluteus medius* and *M. biceps femoris* required combining samples from both sides to produce one rump (*M. gluteus medius*) and one outside (*M. biceps femoris)* sample per carcass. Both oyster blade shoulder cuts had all bones, cartilage and sinews removed, then were rolled and netted (shoulder; AUSMEAT 5050). Subcutaneous fat and epimysium were removed from all cuts collected. These cuts were then vacuum packaged and allocated to a storage period of 5, 21 or 45 days at 2 °C. From each carcass, both loins, topsides and shoulders were allocated in a balanced design to two out of the three storage periods, as described in Table 2. The rump and outside were treated as a “pair” but were also allocated to different storage periods (Table 2). After the allocated storage period, the cuts were prepared for either grill or roast sensory testing. The loin, topside, rump and outside were allocated to grill sensory testing, while the shoulder was allocated to roast sensory testing. For the grill cuts, 5 samples of 15 mm thickness were sliced per cut, vacuum packaged and frozen at −20 °C, whereas the shoulder cuts remained whole and were frozen at −20 °C until subsequent eating quality sessions. After freezing, samples were transported to Texas Tech University (Lubbock, TX, USA), where they remained frozen for subsequent grill or roast sensory testing. All samples were frozen for less than 10 months before they were used for sensory testing.

### 2.3. Sensory Testing

This project was approved by the Murdoch University Human Research Ethics Committee (2016/015) and Texas Tech University Protection of Human Subjects Committee (IRB2017-514). Samples were sensory-tested by untrained consumers in 12 grill (*n* = 720 consumers) and 4 roast (*n* = 240 consumers) sessions, following the Meat Standards Australia sensory testing protocols previously described by Thompson et al. [20] and Watson et al. [21]. For each grill session, five steaks from each cut were cooked, targeting a medium degree of doneness (internal temperature of 65 °C) using a Silex griller (Model S-143K, Silex Grills Australia Pty Ltd., Marrickville, Australia) with temperature set at 180–195 °C, and rested for 45 s. For each roast session, 36 shoulder cuts were cooked simultaneously, removed from the oven (when the internal temperature reached 65 °C) and rested for a minimum of 10 min. The roast cuts were sliced into 4 mm slices, with surrounding fat and gristle seams removed from the slices. Slices were stored in insulated water bath warming units with stainless steel pan inserts set to 60 °C (Model W-3Vi, American Permanent Ware Company, Dallas, TX, USA) to minimise any drying effect and keep the samples warm. For both grill and roast sessions, the steaks or slices were halved (creating 10 samples per cut) before being served. Untrained consumers assessed each sample for tenderness, juiciness, flavour, and overall liking using a 100-score scale, with 100 being the most preferred. All consumers assessed six test samples (three grain and three grass) across the different treatment groups. These six test samples were allocated to the consumers using a Latin square design [20]. Each sensory session consisted of 60 unique consumers testing 36 cuts in total, with each cut of meat being eaten by 10 different consumers, resulting in 10 consumer responses per cut of meat.

### 2.4. Fatty Acids

At 24 h post-mortem, a 20 g sample of loin was collected from each carcass for fatty acid determination. Samples were freeze-dried and then 0.5 g of homogenised dry muscle sample was used for fatty acid extraction based on the method described by O’Fallon et al. [22]. The 0.5 g sample was hydrolysed at 55 °C for 1.5 h with 0.1 mL of internal standard (1.2 g nonadecanoic acid in 100 mL chloroform), 0.7 mL of 10N KOH and 5.3 mL of methanol. The sample was then methylated at 55 °C for 1.5 h with 0.6 mL of 24 N of sulphuric acid. The fatty acid methyl esters were extracted into 1 mL of hexane and then quantified by gas chromatography with flame ionization detection on an Agilent GC-FID 6890 system. A capillary column HP INNOWAX GC column (60 m × 0.25 mm, 0.5 μm) was utilised with hydrogen as the carrier gas. The fatty acid concentrations were reported in mg/100 g of meat [23]. The amounts of major fatty acid groups such as saturated fatty acids, monounsaturated fatty acids, polyunsaturated fatty acids, omega-3 and omega-6 were calculated as the sum of fatty acid profiles from gas chromatography quantification. The levels of long-chain omega-3 polyunsaturated fatty acids including eicosapentaenoic acid (EPA) and docosahexaenoic acid (DHA) were also quantified.

### 2.5. Intramuscular Fat and Objective Tenderness Measurements

For intramuscular fat content, approximately 40 g of diced loin muscle from the caudal (lumbar sacral) region was freeze-dried using a Cuddon FD 1015 freeze dryer (Cuddon Freeze Dry, Blenheim, New Zealand). The percentage of fat in the loin was determined using a near-infrared procedure in a Technicon Infralyser 450 (19 wavelengths), calibrated by chloroform Soxhlet extraction following the procedure described by Perry et al. [24].

For shear force determination, approximately 65 g of loin muscle was vacuum packed for 5 days and then frozen at −20 °C. Samples were then cooked in a plastic bag for 35 min at 71 °C in a water bath before being cooled in running water for 30 min after cooking. From each loin sample, six 1 cm^2^ subsamples were cut and shear force was measured using a Lloyd texture analyser (Model LRX, Lloyd Instruments, Hampshire, UK) with a Warner–Bratzler shear blade fitted as described by Hopkins et al. [25]. The six values were averaged to produce a shear force value per loin sample.

### 2.6. Statistical Analysis

Descriptive statistics (mean, standard deviation, minimum and maximum) for various attributes (intramuscular fat, hot carcass weight, GR tissue depth, shear force, predicted temperature at pH 6 and ultimate pH) of each diet treatment group were outputted using the unadjusted data in R (version 4.2.0). 

Linear mixed effects models (R, version 4.2.0) were used to analyse consumer scores for overall liking, tenderness, juiciness and flavour. The analyses were conducted using the 10 individual consumer scores for each cut. Base models for each sensory trait included the fixed effects for cut (loin, topside, rump, outside, shoulder), storage period (5, 21, 45 days) and dietary treatment (grain, grass). Animal identification, replicate group with the same dietary treatment and consumer identification within consumer session when samples were tasted were included as random terms. All relevant first-order interactions between fixed effects were assessed and non-significant (*p* > 0.05) terms were removed in a stepwise manner. This enabled the significant (*p* < 0.05) differences between treatments to be assessed, with predicted means compared using Tukey’s difference tests (R, version 4.2.0). After establishing the base model for each sensory trait, we then tested each carcass measure, incorporating them individually into the base model as covariates and their interaction with each fixed effect with non-significant (*p* > 0.05) terms removed in a stepwise manner. This was done specifically to assess whether the differences between storage periods or dietary treatment groups could be accounted for by correcting for these carcass measures.

General linear models were used to individually analyse carcass measures and fatty acid profiles as dependent variables and identify differences between the diet treatments. Carcass measurements included intramuscular fat, hot carcass weight, GR tissue depth, shear force, predicted temperature at pH6 and ultimate pH. Measurements taken from the loin were also analysed for their effects on the sensory scores of other cuts, these measures included intramuscular fat, shear force, predicted temperature at pH6 and ultimate pH. Animal identification and consumer identification within the consumer session were included as random terms.

## 3. Results

### 3.1. Carcass Data

The unadjusted carcass measurements of the grain- and grass-fed animals are presented in Table 3. Outcomes of the general linear models indicated differences between the diet treatments for some of these carcass measurements. The grain-fed animals had a higher hot carcass weight (predicted means ± standard error: 30.3 ± 0.05 kg; *p* < 0.05) and deeper GR tissue depth (18.2 ± 0.07 mm; *p* < 0.05) than the grass-fed animals (28.3 ± 0.05 kg; 14.2 ± 0.07 mm). The predicted temperature at pH 6 was also higher in the grain-fed animals (26.5 ± 0.16 °C; *p* < 0.05) than in the grass-fed animals (22.1 ± 0.16 °C). Intramuscular fat of the grain-fed carcasses was higher (4.3 ± 0.02%; *p* < 0.05) compared to that of the grass-fed carcasses (3.9 ± 0.02%), whereas the ultimate pH of the grass-fed carcasses was 0.05 units greater (5.63 ± 0.01; *p* < 0.05) than that of grain-fed carcasses (5.57 ± 0.01). Shear force was not different (*p* > 0.05) between grain- and grass-fed carcasses.

### 3.2. Fatty Acids

The unadjusted mean and standard deviation of the major fatty acid groups of the loin from grass- and grain-fed animals are shown in Table 4. General linear model outcomes showed differences between the diet treatments for the major fatty acid groups, and these are presented below with predicted means (±standard error). There were almost twice as many omega-6 fatty acids in grain-fed lamb samples (802 ± 11.8 mg per 100 g muscle; *p* < 0.05) compared to grass-fed samples (481 ± 11.8 mg per 100 g muscle). Total polyunsaturated fatty acids were also higher in grain-fed lambs (1198 ± 21.3 mg per 100 g muscle; *p* < 0.05) compared to grass-fed lambs (1091 ± 21.3 mg per 100 g muscle), whereas grass-fed samples (510 ± 9.47 mg per 100 g muscle; *p* < 0.05) had almost twice as many omega-3 fatty acids than grain-fed samples (298 ± 9.47 mg per 100 g muscle). Total EPA was higher in the grass-fed samples (98.5 ± 1.82 mg per 100 g muscle; *p* < 0.05) compared to the grain-fed samples (54.7 ± 1.82 mg per 100 g muscle). Likewise, DHA was higher in the grass-fed samples (37.5 ± 0.98 mg per 100 g muscle; *p* < 0.05) than in the grain-fed meat samples (27.7 ± 0.98 mg per 100 g muscle). There was no difference (*p* > 0.05) between the diet treatments for total monounsaturated, saturated and the ratio of polyunsaturated to saturated fatty acids.

### 3.3. The Effect of Diet, Cut and Storage Period on Eating Quality

The outcomes of the base models for each sensory trait are presented in Table 5, and their predicted means (±SE) for cut, diet and storage period for each sensory trait are shown in Table 6. Of the total 5760 observations, the available data represent 5690 eating quality observations from 960 untrained consumers used in the linear mixed effects base models. 

Samples from grain- and grass-fed animals received (*p* > 0.05) similar consumer sensory scores for the same cut after storage for 21 and 45 days (Table 6). However, for the 5-day storage period, the rump and topside samples from grain-fed animals scored 12.4 and 8.9 higher (*p* < 0.05) for overall liking and 13.7 and 11.0 higher for tenderness than their respective grass-fed samples. Likewise, 5-day-aged outside and loin grain-fed samples were both 14.1 scores higher (*p* < 0.05) for tenderness than their grass-fed counterparts. In the overall liking base model, the addition of hot carcass weight, GR tissue depth and the predicted temperature at pH 6 removed the diet effect for the 5-day aged loin, topside, outside and shoulder but not the rump. In the tenderness base model, the addition of GR tissue depth and predicted temperature at pH 6 removed the diet effect for the 5-day-aged rump, topside and outside but not the loin. When the tenderness base model was corrected for shear force, the difference between the grass- and grain-fed outside and shoulder samples aged for 5 days was no longer significant, yet was still significantly different from the results for rump, topside and loin samples (*p* < 0.05). 

Predominantly, higher (*p* < 0.05) sensory scores were observed for samples with extending storage times from 5 to 21 days across most cuts and diets (Table 6). No eating quality improvements (*p* > 0.05) were observed from 21 to 45 days of aging for most cuts. Exceptions to this trend were grain-fed rump, outside, loin and topside samples, which were not different (*p* > 0.05) between 5 and 21 days for overall liking, juiciness or flavour scores. Grass-fed samples also received similar (*p* > 0.05) juiciness (loin, topside) and flavour (loin) scores between 5 and 21 days.

## 4. Discussion

### 4.1. Grass versus Grain Effect

Contrary to our hypothesis, US consumers typically scored grain- and grass-fed lamb meat samples the same, but only after storage for 21 or 45 days. These findings were consistent with those of Pethick et al. [26] and a review by Pethick et al. [27] whereby untrained consumers could not detect sensory differences between pasture- and grain-based diets. However, there is some evidence that trained panellists can detect these differences [15]. Priolo et al. [15] utilised a small number of trained panellists (*n* = 10) for sensory evaluations of lamb but did not score for overall liking or flavour, which limits the capacity to make comparisons with the current study. Within the same study, stall-fed lamb loin samples had higher tenderness and juiciness scores than samples of pasture-fed lambs. In part, this could be due to the higher intramuscular fat content of stall-fed lambs, which was positively correlated with tenderness and juiciness scores [15] and aligns with previous studies showing the positive impact of intramuscular fat on sensory scores [28]. The different sensory responses observed in the current lamb study between diet treatments after 5 days of storage are unlikely to be explained by the differing intramuscular fat levels, which were numerically small but significant (0.4%) and, when included in the base model as a covariate, did not account for any of the difference between grass- and grain-fed samples. Furthermore, samples were denuded of subcutaneous fat, which may have reduced the flavour effect [13]. Therefore, other biological factors must have influenced the sensory score differences between the grass- and grain-fed samples after 5 days of storage.

To explore this further, we tested other carcase phenotype measures within the analysis. Hot carcass weight, GR tissue depth and predicted temperature at pH 6 accounted for the difference between the grass- and grain-fed overall liking scores after 5 days of storage. This likely reflected that the grass-fed carcasses were lighter and possessed less external fat than the grain-fed carcasses, resulting in colder temperatures at pH 6. Therefore, we speculate that the difference between grass- and grain-fed lambs may have been partially due to the impact of cold shortening for some carcasses, the effect of which disappeared with increasing storage time. Under commercial Meat Standards Australia practice, to avoid the effects of cold-shortening, all lamb must be pre-aged in a vacuum for a minimum of 5 days post-slaughter following electrical stimulation or for 10 days without electrical stimulation [29]. As the lambs in the current study were all electrically stimulated post-slaughter, this minimised the likelihood of cold shortening, although some variation between carcasses always exists. 

An alternative explanation for the difference between grass- and grain-fed lamb at 5 days of storage could be the effect of branched-chain fatty acids that differed in concentration between the two diet groups. In particular, pastoral flavours in sheepmeat are mostly determined by branched-chain fatty acids, 3-methylindole (skatole) and the oxidation of linolenic acid and its derivatives [30,31]. Unsaturated linolenic acid (C18:3) is typically higher in meat derived from grass-fed ruminants compared to grain-fed ruminants [32]. The oxidation of linolenic acid and its derivatives can cause the development of pastoral flavours in sheepmeat [33] and unpleasant odours due to the formation of volatile compounds during cooking [34]. In addition, grass feeding reportedly reduces methyl-branched-chain fatty acids yet increases skatole due to a higher ratio of acetate to propionate because of the higher fibre content of the grass compared with the grain diet [30]. Skatole concentration, which is higher in the fat of ruminants finished on grass, is especially of interest as it has been associated with boar taint in pigs [35] and pastoral flavour in sheepmeat [31], and has been referred to as a faecal-smelly compound [30]. These unpleasant characteristics attributed to linolenic acid and skatole may also have contributed to the lower sensory response to the grass-fed samples compared to the grain-fed samples after 5 days of storage. However, storage time beyond 5 days appears to change the sensory response of consumers to grain- and grass-fed meat samples, though the reasons behind this are unclear. It was speculated that additional storage time may have reduced concentrations of skatole and linolenic acid in the samples, which then negated any diet effects. Therefore, the concentration of linolenic acid and skatole in grain- and grass-fed samples across different storage periods deserves further experimentation.

The fatty acid findings were consistent with previous lamb studies where grass-based diets increased muscle omega-3 while grain-based diets increased omega-6 polyunsaturated fatty acid concentrations in the meat [8,36,37], reflecting the fatty acid composition of the diets fed. Depending on grass species, the fatty acid composition varies, typically with 55–70% omega-3 and 10–20% omega-6 [38,39], while grains like barley and maize are abundant in omega-6 and contain minimal amounts of omega-3. Therefore, it can be inferred that grass-fed sheep received a substantial portion of polyunsaturated fatty acid as omega-3 while the grain-fed animals received a higher portion of omega-6 in their diet, which was reflected in the muscle tissue. Similar to Ponnampalam et al. [8], the current lamb study also observed no difference between grass and grain diets for saturated and monounsaturated fatty acid levels in lamb meat, while higher polyunsaturated fatty acid content was found in grain-fed samples compared to grass-fed samples. Ultimately, these fatty acid profiles were consistent with the results of previous literature [8,36,37] and confirm that the grain and grass diets in the current study produced typical fatty acid responses in the meat. 

### 4.2. The Effect of Extended Storage

Contrary to our hypothesis, 5-day-aged samples typically received the lowest sensory scores. Furthermore, increasing storage time from 5 to 21 days generally increased sensory scores, and extending storage from 21 to 45 days resulted in no further change in sensory responses and certainly no deterioration in these scores as we had originally hypothesised. These counter the anecdotal suggestions that US consumers perceive Australian sheepmeat as more “gamey” or “stale” compared to US lamb, partly believed to be due to the extended chilled shipping times contributing to longer-aged meat. Sensory scores were also expected to decrease after 21 days, following observations by Phelps et al. [12], who reported that untrained US consumers (*n* = 360) scored 42-day-aged lamb loins lower than their 21-day counterparts for overall liking, tenderness, juiciness and flavour, although the study did not test animal diet differences. Other studies show that sheepmeat shear force values decreased as storage time post-mortem increased from 12 to 24 days [9] due to the breakdown of protein and connective tissue [9,40]. Therefore, the increased sensory scores observed in the current study as storage time increased from 5 to 21 days likely reflected a similar decline in shear force values due to the breakdown of connective tissue in these samples.

In the US, 47% of the volume of sheepmeat consumed is from loin and leg cuts, followed by the shoulder at 21% [41]. The familiarity of the US consumers with the taste of loin and leg cuts may have contributed to consumers detecting a larger magnitude of differences in sensory scores between grain- and grass-fed samples in these cuts. Although, these differences declined with additional storage time beyond 5 days. In the current study, US consumers scored the roasted shoulder, on average, lower than in other sheepmeat studies [42,43]. This is surprising as the shoulder cut is commonly consumed by US consumers [41], and the cooking method and preparation of shoulder samples to a 4 mm slice thickness were similar to those of Payne et al. [42] and Pethick et al. [43]. However, the current study utilised US consumers, collectively known to be unfamiliar with sheepmeat as a protein [3], whereas Payne et al. [42] and Pethick et al. [43] utilised Australian consumers, who nationally consume over 14 times as much sheepmeat per person per year compared to the US [5]. Further work including leg and rack roasts would improve the understanding of the influence of diet and storage times for different meat cuts using the roast cooking method. Should Australia wish to expand their sheepmeat market to the US, it would be important to address this relationship in future studies.

## 5. Conclusions

These findings demonstrate that US consumers cannot distinguish between grass- and grain-fed lamb. For this reason, grain feeding is not warranted for lamb shipped from Australia by sea to the US to improve consumer sensory perception. Furthermore, increasing storage times from 5 to 21 days improves sensory scores, while further aging from 21 to 45 days maintains eating quality scores. Therefore, storage times beyond 5 days associated with chilled transportation will have no negative impact on Australian lamb eating quality. The diet treatment was also observed to influence total muscle omega-3 and omega-6 concentrations. Measuring skatole and linolenic acid concentrations in grain- and grass-fed samples across different storage periods deserves further experimentation. Additionally, the inclusion of other roast cuts for comparison to the shoulder cut could be beneficial. 

## Figures and Tables

**Table 1:** Mean and standard deviation for dry matter content (DM%) and nutritive characteristics of grass and grain (final phase 50% lupin, 50% barley) dietary treatments.

	Grain	Grass
Total Dry Matter (% DM)	90.3 ± 0.64	28.4 ± 1.90
Moisture (% DM)	9.8 ± 0.64	71.6 ± 1.90
Digestible Dry Matter (% DM)	88.0 ± 4.24	68.3 ± 1.53
* Digestibility of Organic Dry Matter (% DM)	86.0 ± 4.24	64.3 ± 1.53
Metabolisable Energy (MJ/kg DM)	13.3 ± 0.57	10.1 ± 0.14
Crude Protein (% DM)	24.4 ± 15.27	17.8 ± 2.24
Acid Detergent Fibre (% DM)	14.0 ± 12.73	32.3 ± 1.53

* Digestibility of organic dry matter was calculated as the portion of the organic dry matter that could be digested by the animal and is expressed as a percentage of dry matter. This parameter objectively measures the quality of the feed and takes into account the inorganic matter (ash–(sand and dirt)). It is calculated by an industry-agreed equation that relates the digestibility of organic dry matter to digestible dry matter.

**Table 2:** Allocation of cuts from a carcass to storage periods repeated across the 80 carcasses.

Carcass Number	5-Day-Aged	21-Day-Aged	45-Day-Aged
1	Loin		Loin
	Topside	Topside	
		Rumps	Outsides
	Shoulder	Shoulder	
2	Loin	Loin	
		Topside	Topside
	Rumps	Outsides	
		Shoulder	Shoulder
3		Loin	Loin
	Topside		Topside
		Outsides	Rumps
	Shoulder		Shoulder
4	Loin	Loin	
	Topside		Topside
	Outsides	Rumps	
	Shoulder	Shoulder	

**Table 3:** Unadjusted mean ± standard deviation with min–max range in parentheses for intramuscular fat, hot carcass weight, GR tissue depth, shear force, predicted temperature at pH 6 and ultimate pH of grain- and grass-fed carcasses.

Variable	*n*	Grain	*n*	Grass
Intramuscular fat (%)	39	4.3 ± 1.03 (2.6–7.2)	40	3.9 ± 0.88 (2.5–6.3)
Hot carcass weight (kg)	40	30.3 ± 2.91 (22.4–35.7)	39	28.2 ± 2.27 (24.1–35.3)
GR tissue depth (mm)	38	18.5 ± 4.34 (7.5–25.0)	40	14.2 ± 3.33 (7.5–21.0)
Shear force (N)	39	32.8 ± 9.44 (20.0–65.3)	38	33.2 ± 7.85 (23.4–57.4)
Predicted temperature at pH 6	30	26.4 ± 7.34 (6.9–37.1)	27	22.4 ± 8.31 (6.0–37.1)
Ultimate pH	40	5.57 ± 0.07 (5.47–5.78)	40	5.63 ± 0.06 (5.54–5.80)

**Table 4:** Unadjusted mean ± standard deviation for the fatty acid groups (mg/100 g muscle) of the *M. longissimus lumborum* of lamb fed a grass (*n* = 40) or grain (*n* = 40) diet.

	Mean ± Standard Deviation	
Fatty Acid Group	Grain	Grass
ΣPUFA	1197.6 ± 151.3	1090.6 ± 116.2
ΣSFA	3881.5 ± 975.1	3783.9 ± 873.7
ΣPUFA: ΣSFA	0.32:1 ± 0.06	0.30:1 ± 0.06
ΣMUFA	3652.2 ± 974.7	3378.52 ± 874.4
Σn-3	297.9 ± 51.2	510.3 ± 67.5
Σn-6	801.7 ± 93.1	481.3 ± 50.3
EPA	54.7 ± 9.5	98.5 ± 13.2
DHA	27.7 ± 5.2	37.5 ± 7.1

ΣSFA: total saturated fatty acids; ΣMUFA: total monounsaturated fatty acids; ΣPUFA: total polyunsaturated fatty acids; Σn-3: total omega-3 PUFA; Σn-6: total omega-6 PUFA; EPA: eicosapentaenoic acid (C20:5n-3); DHA: docosahexaenoic acid (C22:6n-3). Saturated fatty acids include C8:0, C10:0, C11:0, C12:0, C13:0, C14:0, C15:0, C16:0, C17:0, C18:0, C20:0, C21:0, C22:0, C23:0 and 24:0. Polyunsaturated fatty acids include C18:2n-9trans, C18:2n-6, C18:3n-6, C18:3n-3, C18:4n-3, C20:2n-2, C20:3n-6, C20:4n-6, C20:3n-3, C20:5n-3, C22:2n, C22:4n-6, C22:5n-3 and C22:6n-3. Monounsaturated fatty acids include C14:1, C15:1, C16:1, C17:1, C18:1cis + trans, C20:1, C22:1n-9 and C24:1. Total n-3 fatty acids include C18:3n-3, C18:4n-3, C20:3n-3, C20:5n-3, C22:5n-3 and C22:6n-3. Total n-6 fatty acids include C18:2n-6, C18:3n-6, C20:3n-6, C20:4n-6 and C22:4n-6.

**Table 5:** F-values and numerator degrees of freedom for the effects of the base linear mixed effects models of the overall liking, tenderness, juiciness and flavour sensory scores.

		Sensory Trait, F-Value			
Fixed Effects and Interactions	NDF	Overall Liking	Tenderness	Juiciness	Flavour
Cut	4	88.6	117.7	92.4	64.9
Diet	1	ns	8.0	ns	ns
Storage	2	122.0	369.7	83.0	61.9
Cut × Diet	4	ns	ns	ns	ns
Cut × Storage	8	14.8	27.7	7.4	7.6
Diet × Storage	2	13.9	23.0	9.5	8.7
Cut × Diet × Storage	8	2.7	2.5	ns	2.3

ns: non-significant (*p* > 0.05); NDF: numerator degrees of freedom. Stated F-values indicate significant effects (*p* < 0.05).

**Table 6:** Predicted means (±SE) for cut (rump, outside, loin, topside, shoulder), diet (grain, grass) and storage period (5, 21, 45 days) on overall liking, tenderness, juiciness and flavour sensory scores.

Cut	Storage	Diet	Overall Liking	Tenderness	Juiciness	Flavour
Rump	5	Grain	67.5 ± 2.25 ^b^	66.2 ± 2.44 ^b^	69.9 ± 2.34 ^ab^	64.5 ± 2.22 ^ab^
		Grass	55.1 ± 2.26 ^a^	52.5 ± 2.44 ^a^	59.0 ± 2.34 ^a^	54.7 ± 2.22 ^a^
	21	Grain	69.0 ± 2.26 ^b^	77.6 ± 2.45 ^c^	66.7 ± 2.35 ^ab^	65.5 ± 2.22 ^ab^
		Grass	73.3 ± 2.35 ^b^	78.5 ± 2.44 ^c^	71.8 ± 2.341 ^b^	70.0 ± 2.22 ^b^
	45	Grain	72.4 ± 2.85 ^b^	79.8 ± 3.02 ^c^	68.0 ± 2.95 ^ab^	68.6 ± 2.82 ^b^
		Grass	68.3 ± 2.84 ^b^	78.0 ± 3.01 ^bc^	70.7 ± 2.94 ^ab^	65.9 ± 2.80 ^ab^
Outside	5	Grain	56.0 ± 2.35 ^ab^	51.6 ± 2.53 ^b^	65.0 ± 2.43 ^ab^	56.2 ± 2.31 ^ab^
		Grass	46.6 ± 2.19 ^a^	37.5 ± 2.38 ^a^	56.1 ± 2.27 ^a^	49.5± 2.16 ^a^
	21	Grain	65.0 ± 2.26 ^bc^	69.3 ± 2.45 ^c^	68.1 ± 2.35 ^b^	61.5 ± 2.22 ^b^
		Grass	66.3 ± 2.26 ^bc^	66.1 ± 2.44 ^c^	68.7 ± 2.34 ^b^	65.2 ± 2.22 ^b^
	45	Grain	68.6 ± 2.19 ^c^	71.9 ± 2.38 ^c^	72.1 ± 2.27 ^b^	66.7 ± 2.15 ^b^
		Grass	68.1 ± 2.20 ^c^	69.7 ± 2.39 ^c^	73.0 ± 2.28 ^b^	65.8 ± 2.16 ^b^
Loin	5	Grain	58.2 ± 1.73 ^ab^	61.1 ± 1.92 ^b^	56.0 ± 1.79 ^a^	58.7 ± 1.67 ^ab^
		Grass	52.5 ± 1.73 ^a^	47.0 ± 1.92 ^a^	52.0 ± 1.79 ^a^	54.3 ± 1.68 ^a^
	21	Grain	64.3 ± 1.73 ^bcd^	69.8 ± 1.91 ^c^	56.7 ± 1.78 ^a^	62.5 ± 1.67 ^bc^
		Grass	61.9 ± 1.72 ^bc^	67.0 ± 1.91 ^bc^	56.5 ± 1.78 ^a^	60.6 ± 1.67 ^ab^
	45	Grain	66.6 ± 1.84 ^cd^	73.9 ± 2.03 ^c^	66.9 ± 1.91 ^b^	63.7 ± 1.79 ^bc^
		Grass	70.2 ± 1.69 ^d^	73.8 ± 1.88 ^c^	69.1 ± 1.75 ^b^	68.2 ± 1.63 ^c^
Topside	5	Grain	49.6 ± 1.73 ^b^	42.2 ± 1.92 ^b^	51.5 ± 1.79 ^ab^	52.0 ± 1.67 ^ab^
		Grass	40.7 ± 1.75 ^a^	31.2 ± 1.94 ^a^	48.0 ± 1.81 ^a^	46.2 ± 1.70 ^a^
	21	Grain	56.4 ± 1.69 ^bc^	60.2 ± 1.89 ^c^	56.2 ± 1.76 ^bc^	55.4 ± 1.64 ^bc^
		Grass	56.2 ± 1.72 ^bc^	58.3 ± 1.91 ^c^	54.7 ± 1.78 ^abc^	55.5 ± 1.67 ^bc^
	45	Grain	61.6 ± 1.69 ^c^	63.9 ± 1.88 ^c^	61.4 ± 1.74 ^c^	60.3 ± 1.63 ^c^
		Grass	58.8 ± 1.70 ^c^	61.4 ± 1.89 ^c^	60.2 ± 1.76 ^c^	57.5 ± 1.65 ^bc^
Shoulder	5	Grain	50.6 ± 1.83 ^a^	57.4 ± 1.97 ^a^	50.2 ± 1.85 ^a^	48.9 ± 1.78 ^a^
		Grass	46.9 ± 1.82 ^a^	54.1 ± 1.97 ^a^	45.5 ± 1.85 ^a^	45.1 ± 1.78 ^a^
	21	Grain	49.2 ± 1.85 ^a^	58.7 ± 1.99 ^a^	47.3 ± 1.88 ^a^	47.7 ± 1.81 ^a^
		Grass	45.2 ± 1.82 ^a^	54.0 ± 1.96 ^a^	45.8 ± 1.85 ^a^	44.1 ± 1.78 ^a^
	45	Grain	49.1 ± 1.81 ^a^	59.5 ± 1.95 ^a^	50.9 ± 1.84 ^a^	47.8 ± 1.76 ^a^
		Grass	46.8 ± 1.83 ^a^	58.0 ± 1.97 ^a^	49.1 ± 1.85 ^a^	45.3 ± 1.78 ^a^

For the same cut, letters that differ indicate a significant difference within columns for overall liking, tenderness, juiciness and flavour (*p* < 0.05).

## Data Availability

All related data and methods are presented in this paper. Additional inquiries should be addressed to the corresponding author.
